# Severe Erosive Pill Esophagitis Induced by Crizotinib Therapy: A Case Report and Literature Review

**DOI:** 10.1155/2016/3562820

**Published:** 2016-12-08

**Authors:** Patrick Jung, Kyle J. Fortinsky, Zane R. Gallinger, Piero Tartaro

**Affiliations:** ^1^Department of Internal Medicine, University of Toronto, Toronto, ON, Canada; ^2^Division of Gastroenterology, University of Toronto, Toronto, ON, Canada

## Abstract

Previous case reports have described esophagitis thought to be secondary to crizotinib, an oral tyrosine-kinase inhibitor used in the treatment of anaplastic lymphoma kinase- (ALK-) positive non-small cell lung cancer (NSCLC). In those reports, the interval development of esophagitis was between two days and three months after initiating or reinitiating crizotinib therapy. We present a woman who developed ulcerative esophagitis ten months after beginning crizotinib therapy, which is highly unusual. We believe the provoking factor was a change in her medication administration routine, done to accommodate religious practices during the period of Ramadan. This case illustrates the mechanism of pill esophagitis and reinforces the importance of patient education when it comes to medication administration. Clinicians may consider early imaging or investigations in patients with concerning symptomatology in the context of crizotinib therapy or other offending medications. Future research may help to uncover additional risk factors for this exceedingly rare diagnosis in this patient population. Most importantly, this case highlights nonpharmacologic ways to improve tolerability and decrease adverse effects of a highly effective chemotherapeutic agent.

## 1. Introduction

The oral tyrosine-kinase inhibitor, crizotinib, has been approved for use in locally advanced or metastatic non-small cell lung cancer (NSCLC) that is anaplastic lymphoma kinase- (ALK-) positive. The phase III trial completed prior to approval noted visual disturbances and gastrointestinal symptoms (e.g., nausea, vomiting, and diarrhea) as common adverse events, but no cases of esophagitis were reported [1]. Six case reports have since been published in English describing esophagitis suspected to be secondary to crizotinib [2–7]. In these reports, esophagitis appears to have developed two days to three months after initiating or reinitiating crizotinib therapy. We present a woman who developed acute odynophagia and severe erosive esophagitis ten months after beginning crizotinib.

## 2. Case Presentation

A 45-year-old female, lifelong nonsmoker, was admitted with a one-day history of severe odynophagia preventing any oral intake. Her past medical history was significant for stage IV ALK-positive NSCLC, with metastases to the pleura and local lymph nodes, in situ ductal carcinoma of the left breast, for which the patient had opted for surveillance rather than lumpectomy, and irritable bowel syndrome. She had been on crizotinib 250 mg by mouth twice daily for ten months prior to presentation. Her only other medication was acetaminophen 500 mg by mouth once daily. She was not on any nonsteroidal anti-inflammatory drugs (NSAIDs) and she denied taking any nonprescription medications or herbal supplements. There had been no recent changes to her medications. She had received palliative radiotherapy to the primary lung mass three months earlier.

The patient stated that she had been fasting from sunrise to sunset for the previous month in observance of Ramadan. Consequently, her medication administration routine had changed. She was taking her evening dose of crizotinib later than usual (i.e., at 21:00 rather than 19:00 hours) immediately before bedtime and her morning dose earlier than usual (i.e., at 03:30 rather than 07:00 hours, to ensure she was not breaking her fast during daylight hours) before returning to sleep. Therefore, the patient lay supine immediately after taking each dose of crizotinib.

**Figure 1:**
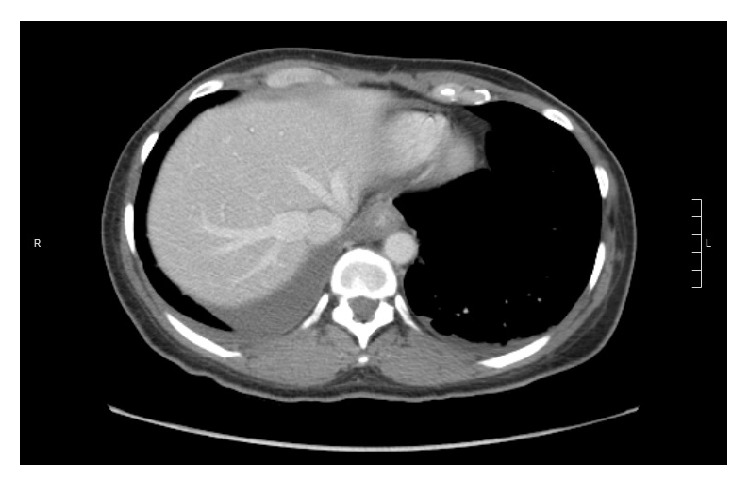
Abdominal CT scan revealing thickening of the distal esophagus.

**Figure 2:**
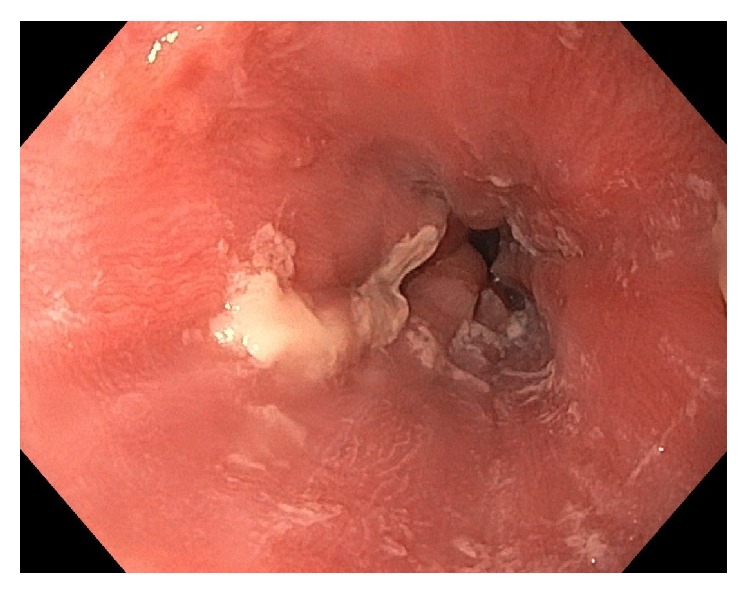
Endoscopic image of esophagitis extending to the gastroesophageal junction at 40 cm.

**Figure 3:**
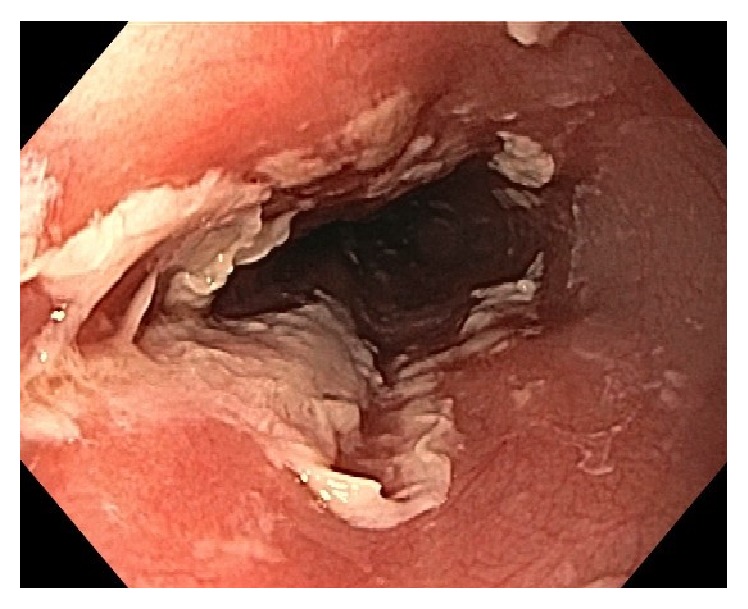
Endoscopic image of extensive ulcerative esophagitis in the mid-esophagus.

**Figure 4:**
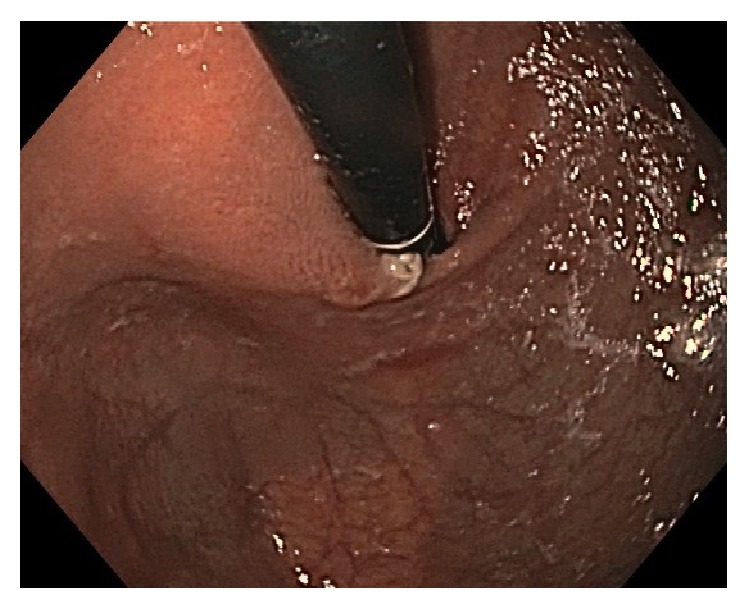
Endoscopic image of the gastric fundus on retroflexion showing evidence of esophagitis and normal surrounding stomach mucosa.

After 14 days of this altered medication regimen, the patient presented to our Emergency Department complaining of odynophagia and inability to tolerate any oral intake for the last 24 hours. Blood work, electrocardiogram, and chest X-ray were unremarkable. The patient was admitted to hospital for further workup. Upon admission to hospital, a CT scan of the chest and abdomen showed thickening of the distal esophagus (Figure 1). Esophagogastroduodenoscopy was performed the following day and revealed ulcerative esophagitis at the mid-esophagus at 15 cm and at the gastroesophageal junction at 40 cm (Figures 2, 3, and 4). The findings on endoscopy were most consistent with pill esophagitis. The findings were not typical of CMV, HSV, or candida infection.

Given the patient's clinical symptoms and the severity of endoscopic findings, we liaised with the patient's medical oncologist and decided to hold the crizotinib and to start the patient on pantoprazole 40 mg by mouth twice daily and sucralfate 1 g by mouth twice daily. Her odynophagia improved within three days of stopping crizotinib, and the patient was discharged home four days after admission being able to tolerate a regular diet without any difficulty. After discussion with the patient's medical oncologist, the crizotinib was permanently discontinued as the patient's CT scans demonstrated tumor progression despite therapy. The patient was then switched to second-line ceritinib. Pantoprazole and sucralfate were stopped after 8 weeks of therapy and there had been no recurrence of her esophageal symptoms at 6-month follow-up.

## 3. Discussion

This appears to be the 7th published case of esophagitis related to crizotinib therapy and the first case that occurred beyond the third month of initiating or reinitiating crizotinib (Table 1). Uniquely, in our case, the patient tolerated crizotinib well for approximately nine months of treatment. The factor that seemed to provoke her severe esophagitis was the change in routine of medication administration. Specifically, the patient changed the time at which she took her medication in order to ensure that she was fasting between sunrise and sunset during the period of Ramadan. This change led to the patient laying supine immediately after taking her crizotinib. Without a preceding history of reflux, dyspepsia, or other offending medications, her presentation is most consistent with pill esophagitis.

Pill esophagitis has been described since the 1970s. The most recognized causative agents include acetylsalicylic acid and nonsteroidal anti-inflammatories, tetracycline antibiotics, potassium chloride, iron compounds, quinidine, and bisphosphonates [8]. Risk factors for pill esophagitis include patient positioning, medication size, inadequate fluid intake with medication administration, and altered esophageal anatomy. These factors may all prolong esophageal transit time, thereby increasing the direct caustic contact between pill and esophageal mucosa. This often results in the typical endoscopic findings of erythema or inflammation, erosions, and areas of ulceration [9].

Not surprisingly, in two of the previous case reports, recurrence of esophagitis was prevented by advising patients to position themselves upright when taking crizotinib and for thirty minutes afterwards [2, 6]. In our case, restarting the crizotinib was not appropriate as our patient had radiographic evidence of tumor burden progression. Fortunately, consistent with previous reports, our patient's symptoms resolved with discontinuation of the crizotinib and the start of pantoprazole and sucralfate.

In summary, this case represents an episode of severe esophagitis secondary to crizotinib therapy for ALK-positive NSCLC, which was provoked by a change in medication administration routine carried out by the patient to accommodate her religious practices. Clinicians should discuss strategies, such as remaining upright for a period of time after medication administration, to reduce the development of pill esophagitis. This case highlights certain ways to improve the tolerability and safety of a highly effective treatment for an increasingly common type of cancer.

## Figures and Tables

**Table 1:** Timing of onset and resolution of symptoms after initiating and discontinuing crizotinib therapy in six previous case reports.

Case report	Onset of symptoms	Resolution of symptoms
Park et al. [2]	Case 1: 3 months after initiation	Case 1: within 28 days
	Case 2: 2 months after initiation	Case 2: within 14 days
Srivastava et al. [3]	2 weeks after initiation	Within 10 days
Takakuwa et al. [4]	2 days after initiation	Not specified
Abdel Jalil et al. [5]	1 week after initiation	Within 14 days
Conduit et al. [6]	1 month after reinitiation	Within 7 days
Tsukita et al. [7]	1 week after initiation, 2 weeks after reinitiation	Within 10 days
